# Editorial: Insights and implications of mechanisms shaping glioma immune landscape, immunotherapy resistance, and relevant translational research

**DOI:** 10.3389/fonc.2024.1485761

**Published:** 2024-09-26

**Authors:** Xiangqi Meng, Linlu Tian, Jinquan Cai

**Affiliations:** ^1^ Department of Neurosurgery, The Second Affiliated Hospital of Harbin Medical University, Harbin, China; ^2^ Department of Immunology, Harbin Medical University, Harbin, China

**Keywords:** glioma, glioma targeting therapy, immune microenvironment, immunotherapy, glioma therapeutic strategies

Research into the glioma immune microenvironment has become a pivotal area of study, driven by the necessity to elucidate the complex interactions between glioma cells and the immune system. Gliomas, particularly glioblastomas, present a significant therapeutic challenge due to their aggressive nature and the intricate mechanisms by which they evade immune surveillance. Advances in this field are essential for developing novel therapeutic strategies capable of improving patient outcomes and addressing the prevalent issue of resistance to current treatments.

Recent investigations have provided valuable insights into the mechanisms underlying glioma-induced immunosuppression. Glioblastomas are characterized by a highly immunosuppressive microenvironment that facilitates tumor growth and impedes effective immune responses. This microenvironment is shaped by a variety of factors, including immune checkpoint molecules, tumor-associated antigens, and cytokines, all of which contribute to the tumor’s ability to evade immune detection and resist conventional therapies.

Emerging research highlights several key immune-related biomarkers and factors associated with glioma progression. For instance, BTN3A1, an immune checkpoint molecule, has been implicated in the aggressive behavior of glioblastomas (Kone et al.). Elevated BTN3A1 expression correlates with a poor prognosis, likely due to its role in modulating immune cell activity and fostering an environment conducive to tumor advancement. The association between BTN3A1 and increased infiltration of immune cells, such as B cells, CD8+ T cells, and M2 macrophages, further underscores its potential as a therapeutic target. These findings suggest that targeting BTN3A1 could enhance therapeutic efficacy and address some of the current limitations in glioma treatment.

Similarly, CLEC7A has emerged as a critical factor in gliomas, particularly concerning tumor progression and immune modulation (Wang et al.). CLEC7A, also known as Dectin-1, is involved in pathogen recognition and immune response regulation. Its expression has been linked to glioma severity and patient prognosis, positioning it as a valuable prognostic biomarker. Additionally, CLEC7A’s role in macrophage function highlights the potential benefits of targeting this protein in immunotherapy strategies. However, further research is required to fully understand CLEC7A’s impact on immune responses within the glioma microenvironment and to translate these insights into clinical practice.

Bibliometric analyses of glioma immunotherapy research reveal a significant focus on immune-checkpoint inhibitors (ICIs) (Yuan et al.). ICIs, including those targeting PD-1/PD-L1 and CTLA-4, have demonstrated considerable promise in various malignancies, and their application in gliomas is an area of active exploration. These analyses provide a comprehensive overview of key contributors, including authors, journals, and institutions, and underscore the need for continued investigation into the challenges associated with ICI therapies, such as resistance mechanisms and optimal treatment combinations.

The application of multi-omics technologies has further advanced the identification of therapeutic targets in gliomas (Pandey et al.). In the context of diffuse intrinsic pontine glioma (DIPG), multi-omics approaches have identified a range of targetable antigens and cell surface proteins, such as CD276 and HER2. These findings offer potential avenues for developing targeted therapies, including chimeric antigen receptor (CAR) and T cell receptor (TCR) therapies. By integrating genomics, proteomics, and immunopeptidomics, researchers are able to uncover novel targets that could be exploited to address the unique characteristics of DIPG and other gliomas.

Additionally, novel therapeutic agents like RRx-001 are being evaluated for their potential to enhance the efficacy of standard glioblastoma treatments (Fine et al.). RRx-001, an NLRP3 inhibitor and nitric oxide (NO) donor, has demonstrated capabilities in preclinical models to sensitize tumors to chemoradiotherapy, normalize tumor vasculature, and repolarize macrophages. This multifaceted approach addresses key limitations of current treatments, including resistance to chemotherapy and radiotherapy. The ongoing clinical trials are critical to determining RRx-001’s safety and efficacy, with the potential to represent a significant advancement in glioblastoma therapy.

In summary, the integration of recent research findings into our understanding of the glioma immune microenvironment and the development of innovative therapeutic strategies are critical for advancing glioma treatment. Continued research and interdisciplinary collaboration are essential to translating these discoveries into effective and personalized therapies. As the field evolves, these advancements hold promise for significantly improving the management of gliomas and enhancing patient outcomes.

